# Influence of Controlled Stomatognathic Motor Activity on Sway, Control and Stability of the Center of Mass During Dynamic Steady-State Balance—An Uncontrolled Manifold Analysis

**DOI:** 10.3389/fnhum.2022.868828

**Published:** 2022-03-25

**Authors:** Cagla Fadillioglu, Lisa Kanus, Felix Möhler, Steffen Ringhof, Daniel Hellmann, Thorsten Stein

**Affiliations:** ^1^BioMotion Center, Institute of Sports and Sports Science, Karlsruhe Institute of Technology, Karlsruhe, Germany; ^2^Department of Prosthodontics, University of Würzburg, Würzburg, Germany; ^3^Department of Sport and Sport Science, University of Freiburg, Freiburg, Germany; ^4^Dental Academy for Continuing Professional Development, Karlsruhe, Germany

**Keywords:** jaw clenching, tongue pressing, masseter, Posturomed, postural control, UCM, covariation, detrended fluctuation analysis

## Abstract

Multiple sensory signals from visual, somatosensory and vestibular systems are used for human postural control. To maintain postural stability, the central nervous system keeps the center of mass (CoM) within the base of support. The influence of the stomatognathic motor system on postural control has been established under static conditions, but it has not yet been investigated during dynamic steady-state balance. The purpose of the study was to investigate the effects of controlled stomatognathic motor activity on the control and stability of the CoM during dynamic steady-state balance. A total of 48 physically active and healthy adults were assigned to three groups with different stomatognathic motor conditions: jaw clenching, tongue pressing and habitual stomatognathic behavior. Dynamic steady-state balance was assessed using an oscillating platform and the kinematic data were collected with a 3D motion capturing system. The path length (PL) of the 3D CoM trajectory was used for quantifying CoM sway. Temporal dynamics of the CoM movement was assessed with a detrended fluctuation analysis (DFA). An uncontrolled manifold (UCM) analysis was applied to assess the stability and control of the CoM with a subject-specific anthropometric 3D model. The statistical analysis revealed that the groups did not differ significantly in PL, DFA scaling exponents or UCM parameters. The results indicated that deliberate jaw clenching or tongue pressing did not seem to affect the sway, control or stability of the CoM on an oscillating platform significantly. Because of the task-specificity of balance, further research investigating the effects of stomatognathic motor activities on dynamic steady-state balance with different movement tasks are needed. Additionally, further analysis by use of muscle synergies or co-contractions may reveal effects on the level of muscles, which were not visible on the level of kinematics. This study can contribute to the understanding of postural control mechanisms, particularly in relation to stomatognathic motor activities and under dynamic conditions.

## Introduction

Balance maintenance and proper body orientation in space are essential for human life. They require a good, reliable and flexible postural control system which is capable of processing multiple sensory feedback inputs from the visual, somatosensory and vestibular systems in the spinal and supraspinal structures of the central nervous system (CNS) in a task dependent manner (Takakusaki, 2017). The control of posture involves control of the body position in space for stability and orientation. Stability is defined as the control of the center of mass (CoM) in relation to the base of support, whereas orientation refers to the ability to maintain an appropriate relationship between the body segments as well as between the body and the environment (Shumway-Cook and Woollacott, 2017). A healthy motor control system modulates the postural movements continuously as a function of the changing tasks. The inability to modulate postural sway, but also environmental or individual constraints may lead to poor performance, instability and falls (Haddad et al., 2013). Furthermore, it has been shown that improved postural control is associated with a decreased risk of falls (Horak, 2006; Rubenstein, 2006) as well as a decreased risk of injury (Hrysomallis, 2007).

Attentional processing is required during postural tasks; therefore, they may reduce the performance of a secondary task when performed simultaneously. On the other hand, a secondary task may improve the postural control by an improved automaticity, an increased arousal or through the utilization of reduced sway for the sake of a better supra-postural task performance (Shumway-Cook and Woollacott, 2017). Previous studies showed that postural control may be influenced by several factors, including motor activity in the stomatognathic motor system (Julià-Sánchez et al., 2020). A frequently cited explanation for this is based on the stimulation of periodontal mechano-receptors that are centrally integrated along with other sensory input and, therefore, facilitates the excitability of the human motor system (Boroojerdi et al., 2000) in a manner similar to the Jendrassik maneuver (Jendrassik, 1885), which in turn increases the neural drive to the distal muscles (Ebben, 2006; Ebben et al., 2008). A variety of studies indicated that stomatognathic motor activity in the form of chewing, tongue activity or different clenching conditions affects human balance and posture under static conditions (Gangloff et al., 2000; Sakaguchi et al., 2007; Hellmann et al., 2011, 2015; Alghadir et al., 2015; Ringhof et al., 2015a,b). Among others, a reduced body sway in the anterior-posterior direction (Hellmann et al., 2011), a reduced variability of muscular co-contraction patterns of posture-relevant muscles of the lower extremities (Hellmann et al., 2015), and reduced trunk and head sway under the influence of controlled biting activities were reported during upright standing (Ringhof et al., 2015a). Furthermore, the review by de Souza et al. (2021) reported that jaw clenching during activities that involve the lower and upper limbs enhance neuromotor stimulation in terms of increased H-reflexes (Miyahara et al., 1996) and stimulate a larger area of the brain. Specifically, a large amount of activity was observed over the frontal, parietal, and temporal cortices and cerebellum during hand grip combined with jaw clenching compared to without jaw clenching (Kawakubo et al., 2014). The authors suggested that the stomatognathic motor system may have effects on the function of remote muscles via cortical activations. Furthermore, a higher excitability of the human motor system during voluntary jaw clenching has also been shown (Boroojerdi et al., 2000). From an evolutionary perspective, it was hypothesized that jaw clenching increases the blood flow to anterior temporal lobe structures during acute activation of the limbic fear circuits (Bracha et al., 2005a). Jaw clenching may increase the blood flow to temporal lobe structures by pumping blood through the temporal bone emissary veins, thus conferring a possible survival advantage during activation of the limbic fear-circuits in expectation of situations requiring the freeze, flight, fight, fright acute fear response (Bracha et al., 2005b). Stomatognathic motor activity also seems to be part of a common physiological repertoire used to improve motor performance during balance recovery tasks (Ringhof et al., 2016). Besides all these facts, it should be mentioned that it stomatognathic motor activity might be of clinical relevance for the prevention of falls. In elderly people there is evidence for an increased risk of falling resulting from an insufficient dental or prosthetic status (Okubo et al., 2010; Mochida et al., 2018).

In contrast to balance under static conditions (e.g., sitting or standing), the influence of stomatognathic motor activity under dynamic conditions (e.g., standing on a balance board or on an oscillating platform) has not yet been investigated in detail (Ringhof et al., 2016). Since the effects found during one balance task may not necessarily be transferable to another balance task (Giboin et al., 2015; Kümmel et al., 2016; Ringhof and Stein, 2018), the question arises whether the effects of stomatognathic motor activity found during static balance tasks would also be observable during dynamic ones. Accordingly, we started to investigate the effects of stomatognathic motor activity in the form of jaw clenching and tongue pressing on dynamic reactive balance performance (Fadillioglu et al., 2022). This was realized by use of an oscillating platform perturbed randomly in one of four horizontal directions. In our previous study, the focus was on the first reactive part of the task. We showed that jaw clenching improved dynamic reactive balance in a task-specific (i.e., direction-dependent) way. The performance improvements found for jaw clenching were associated with lower mean speeds of distinct anatomical regions compared to both the tongue pressing and habitual groups. Subsequent to these findings, the question arises as to whether this performance increase is associated with a changed sway, stability or control of the CoM in the steady-state phase of the task.

The CoM is suggested to be the controlled variable in postural studies (Winter et al., 1998; Kilby et al., 2015; Nicolai and Audiffren, 2019; Richmond et al., 2021), although experimental verification is difficult (Shumway-Cook and Woollacott, 2017). Scholz et al. (2007) used an uncontrolled manifold (UCM) approach to determine if the CoM is the variable which is primarily controlled by the CNS during postural control. They showed that during recovery from a loss of balance, the participants tend to re-establish the position of the CoM rather than those of the joint configurations (Shumway-Cook and Woollacott, 2017), and therefore suggested that the CoM is the key variable controlled by the CNS. In postural control studies, CoM sway is an important parameter (Richmond et al., 2021) and its spatial dynamics can be quantified among others by the total distance covered (Prieto et al., 1996; Richmond et al., 2021). Another important aspect is the temporal dynamics of the sway, since variations in supra-postural activities may lead not only to spatial but also to temporal changes (Chen and Stoffregen, 2012). It was suggested that a detrended fluctuation analysis (DFA) can reveal the temporal dynamics of postural data, specifically to quantify the long-range correlations (or fractality) of the data (Duarte and Sternad, 2008; Stergiou, 2016).

When controlling the body during balance tasks, the CNS has to coordinate a redundant musculoskeletal system (Bernstein, 1967) possessing more degrees of freedom than necessary to achieve the given task (Latash et al., 2002). Different approaches have been suggested to analyze how the CNS treats this redundancy, such as motor programs (Schmidt et al., 2018), optimal control (Todorov and Jordan, 2002) or synergies (d'Avella et al., 2003; Latash et al., 2007; Stetter et al., 2020). Latash et al. (2007) define “synergy” as a neural organization consisting of a multi-element system that organizes sharing of a task among a set of elemental variables (EVs), and ensures the stabilization of a performance variable (PV) through the co-variation of EVs. The fact that different combinations of EVs may result in the same PV indicates that the co-varied behavior provides flexibility for the system. In this context, redundancy is considered not as a problem but as an advantage for the motor control system. According to the motor abundance principle (Gelfand and Latash, 1998), redundancy in the motor control system can be considered positive since the co-variation at the level of the EVs may provide robustness against perturbations (Gera et al., 2010).

The UCM approach (Scholz and Schöner, 1999) is one possibility to quantify the amount of equivalent movement solutions and the degree of stability of the PV. The UCM approach requires a model that relates the changes in EVs to changes in the PV; and ultimately the effects of changes in EVs on the PV are analyzed (Scholz and Schöner, 2014). Both the EVs and PV are chosen on a physiological basis with task-specific considerations. The variability in EVs that results in a changed PV is quantified by the *UCM*_⊥_ component, whereas it is associated with the *UCM*_∥_ component if the PV remains the same even if the EVs vary over repetitions (Scholz and Schöner, 1999; Latash et al., 2007). The UCM approach has been applied to analyze various motor tasks; for example, reaching and pointing (Tseng et al., 2002; Domkin et al., 2005), pistol shooting (Scholz et al., 2000), sit-to-stand (Scholz et al., 2001; Reisman et al., 2002), parkour jumps (Maldonado et al., 2018), treadmill walking (Verrel et al., 2010; Qu, 2012) and running (Möhler et al., 2019). Kinematic or kinetic data were commonly used as EVs to investigate their effects on the PVs. There is also a number of studies that apply UCM to postural tasks (Krishnamoorthy et al., 2005; Freitas et al., 2006; Hsu et al., 2007, 2013; Hagio et al., 2020). When analyzing postural tasks, the CoM is typically chosen as the PV and joint angles as EVs (Krishnamoorthy et al., 2005; Freitas et al., 2006; Hsu et al., 2007, 2013; Scholz et al., 2007; Hagio et al., 2020). By means of UCM analysis, changes in the variability of coordinated joint movements in association with the stability and control of the CoM have been investigated for various setups with different research questions. In line with the previous studies, a UCM analysis was conducted in this study with stomatognathic motor conditions as the independent variable.

The aim of this study was to investigate the influence of different stomatognathic motor activities (jaw clenching and tongue pressing) on the sway, stability and control of the CoM during a dynamic steady-state balance task (one-legged standing on an oscillatory platform after perturbation). The path length (PL) of the 3D CoM was used to quantify the possible effects of different stomatognathic motor activities on the spatial dynamics of CoM sway, whereas its temporal dynamics was assessed with a DFA. A UCM approach was applied to investigate if and how the co-variation of the joint movements led to the stabilization and control of the CoM, which were quantified by *UCM*_*Ratio*_ and *UCM*_⊥_, respectively. Following the results of our above-mentioned study on the influence of jaw clenching and tongue pressing on dynamic reactive balance performance (Fadillioglu et al., 2022), it was hypothesized that these activities decrease the sway and increase the control and stability of the CoM. Therefore, a decreased PL of the CoM trajectory, an increased alpha of DFA, an increased *UCM*_*Ratio*_ and a decreased *UCM*_⊥_ for the jaw clenching group (JAW) and the tongue pressing group (TON) compared to the group with habitual stomatognathic behavior (HAB) were expected. The findings of this study may contribute to the understanding of postural control, particularly in relation to stomatognathic motor activities and under dynamic conditions.

## Methods

This study comprised a follow-on analysis of the original data set used in Fadillioglu et al. (2022). In the previous study, the reactive phase of the task was analyzed, whereas in the present one, the following steady-state phase is investigated. An a priori power analysis was performed based on the findings of the study (Ringhof et al., 2015a) which analyzed the effects of submaximum jaw clenching on postural stability and on the kinematics of the trunk and head. The analysis revealed that 16 participants per group would be enough to reach the sufficient power (>0.8).

### Participants

Forty-eight healthy adults (25 female, 23 male; age: 23.8 ± 2.5 years; height: 1.73 ± 0.09 m; body mass: 69.2 ± 11.4 kg) voluntarily participated in the study after giving written informed consent. All participants completed a questionnaire, confirmed that they were physically active (physical activity 4.6 ± 1.5 days/week and 436 ± 247 min/week) and naive to the tasks on an oscillating platform; had no muscular or neurological diseases; no signs and symptoms of temporomandibular disorders (assessed by means of the RDC/TMD criteria, Dworkin and LeResche, 1992). They presented in good oral health with full dentition (except for 3rd molars) in neutral occlusion. The study was approved by the Ethics Committee of the Karlsruhe Institute of Technology.

### Experimental Procedure

#### Balance Tasks

**Figure 1:**
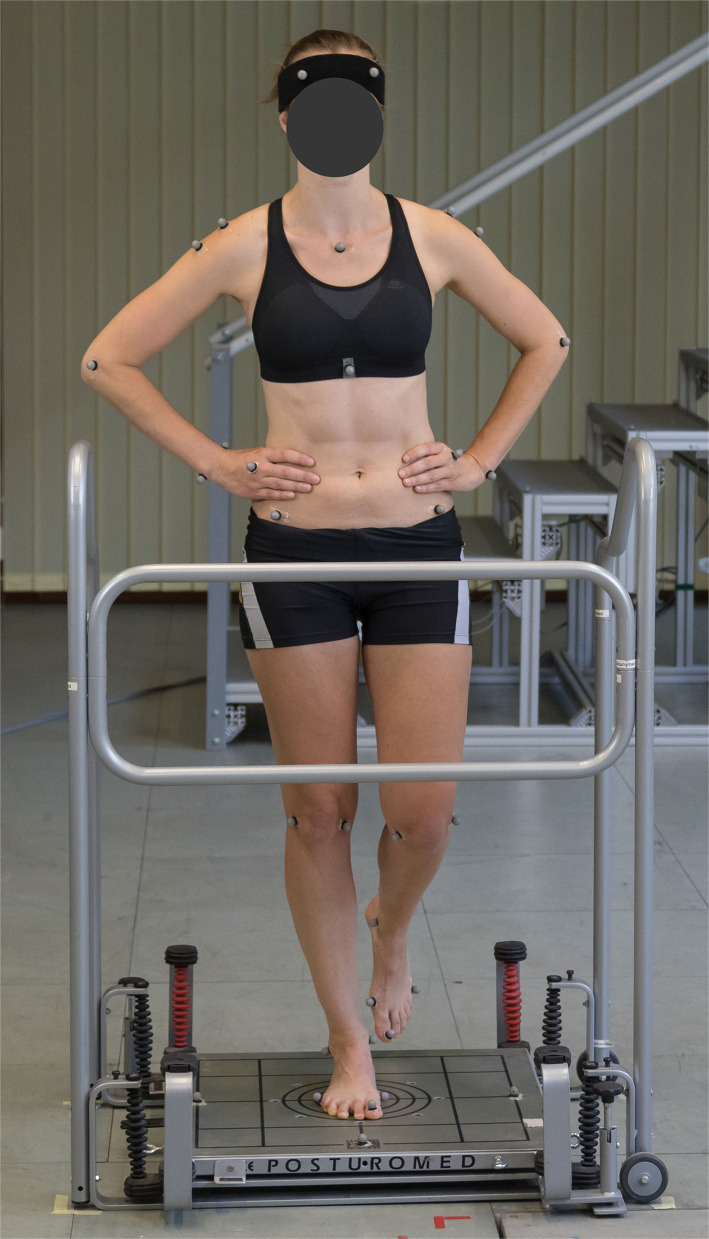
Participant during single-leg stand on the Posturomed oscillating platform.

Dynamic steady-state balance was assessed by means of a Posturomed oscillating platform (Haider-Bioswing, Weiden, Germany), which is a widely-used commercial device to analyze or improve dynamic balance in scientific studies as well as in physiotherapy (Kiss, 2011a; Freyler et al., 2015). It consists of a rigid platform (12 kg, 60 × 60 cm) connected to the main frame by eight 15 cm steel springs with identical stiffness and it can swing in the horizontal plane in all directions. In this study, an automatic custom-made release system was used to initiate mechanical perturbations in four different directions: back (B), front (F), left (L), right (R) (Fadillioglu et al., 2022). By convention, these directions indicate in which direction the platform was accelerated after release of the platform. In each trial, a perturbation in one of the four possible directions was applied in a randomized order. Participants stood on the platform on their dominant leg, which were determined based on self-reports. If the participants were not sure which leg was their dominant leg, it was determined by means of test trials on the Posturomed before the measurements (Ringhof and Stein, 2018). During single-leg stand, they kept their hands placed at the hips and their eyes focused on a target positioned at eye level and 4 m away from the center of the Posturomed (Figure 1).

#### Group Assignment and Masticatory System Statuses

For familiarization, each participant performed two trials without and two trials with perturbation on the Posturomed. After that, a baseline measurement with perturbation and in habitual stomatognathic motor condition was conducted to determine the initial balance performance quantified by Lehr's damping ratio (DR) (Kiss, 2011a). Based on both balance performance and gender, each participant was assigned to one of three groups, such that both gender and the initial level of performance of the groups were balanced. The statistical examination by a one-way ANOVA revealed no baseline performance differences between the three groups (*p* = 0.767). Each group (JAW, TON and HAB) consisted of 16 participants and performed one of the stomatognathic motor conditions simultaneously with the balance task during the measurements (Table 1).

**Table 1:** Stomatognathic motor conditions of the three groups, JAW, TON, and HAB.

**JAW:** instructed, controlled submaximal jaw clenching with a 75 N force—activity of the masticatory muscles during simultaneous occlusal loading
**TON:** instructed, controlled submaximal tongue pressing against the palate—stomatognathic muscle activity without occlusal loading
**HAB:** habitual stomatognathic behavior—jaw positioning without any instruction

The stomatognathic motor activity was recorded by an EMG system (detailed information in the Section Data Collection). To ensure that a force of 75 N was consistently applied, the participants in the JAW group trained just before data acquisition with a RehaBite^®^ (Plastyle GmbH, Uttenreuth, Germany). This medical training device with liquid-filled plastic pads works based on hydrostatic principles, and can be used to control the applied force (Giannakopoulos et al., 2018b). As the participants trained with the RehaBite^®^, masseter activity was monitored to determine the corresponding muscle activity level associated with a jaw clenching force of 75 N. The determined masseter activity level was around 5% maximum voluntary contraction (MVC) for all participants and was used to control if the submaximal jaw clenching condition was fulfilled.

The TON group similarly trained to apply a submaximal force with the tip of the tongue against the anterior hard palate corresponding to an EMG activity level of the suprahyoid muscles of the floor of the mouth of 5% MVC. Both groups trained for the stomatognathic motor task for 5 min. The HAB did not receive any training or instructions. During the measurements, the JAW group performed the jaw clenching task on an Aqualizer^®^ intraoral splint (medium volume; Dentrade International, Cologne, Germany).

### Data Collection

A 3D motion capture system (Vicon Motion Systems; Oxford Metrics Group, Oxford, UK; 10 Vantage V8 and 6 Vero V2.2 cameras with a recording frequency of 200 Hz) was used to record the movements of the Posturomed platform and the participants. Four reflective markers were attached on the upper surface of the Posturomed platform. A further 42 reflective markers were attached to the participants' skin in accordance with the ALASKA modeling system (Advanced Lagrangian Solver in kinetic Analysis, Insys GmbH, Chemnitz, Germany; Härtel and Hermsdorf, 2006). Before data acquisition, 22 anthropometric measures were manually taken from each participant for the ALASKA modeling.

A wireless EMG system (Noraxon, Scottsdale, USA; recording frequency of 2,000 Hz) was used to measure EMG activity of the masseter for JAW and HAB; and of the suprahyoid muscles of the floor of the mouth measured in the region of the digastricus venter anterior muscle for TON. As preparation, the skin over the corresponding muscles was carefully shaved, abraded and rinsed with alcohol. Bipolar Ag/AgCl surface electrodes (diameter 14 mm, center-to-center distance 20 mm; Noraxon Dual Electrodes, Noraxon, Scottsdale, USA) were positioned in accordance with the European Recommendations for Surface Electromyography (Hermens et al., 1999). Afterwards, MVC tests were performed.

For each trial, participants received standardized instruction about the task to be performed and were asked to compensate the perturbation as quickly as possible and to stabilize their body. Between each trial, participants had 2 min of rest to prevent fatigue. Measurements ended after completion of 12 successful balance task trials (i.e., three trials for each direction) each lasting 20 s after initiation of the perturbation. During the measurements, EMG activity of the masseter (for the JAW group) or the suprahyoid muscles of the floor of the mouth (for the TON group) was monitored and compared with the individually determined EMG activity level corresponding to 5% of MVC. Recordings were stopped and the trial was considered invalid if participants stopped performing their stomatognathic motor task (JAW and TON), had ground contact with the non-standing foot, changed the placement of their standing foot, released one of the hands from the hip or lost their balance. All data were recorded in Vicon Nexus 2.10; where the EMG system was connected via the Noraxon plug-in.

### Data Analysis

The collected data of 576 trials (48 participants × three valid trials × four directions) were exported from Vicon Nexus for further analysis in MATLAB R2020a (MathWorks; Natick, USA). For all data, the R and L directions were re-sorted as ipsilateral (I) and contralateral (C) according to the standing leg of the participants.

The marker data were filtered with a Butterworth low-pass filter (fourth-order; cut-off frequency 10 Hz); and the EMG data with a Butterworth band-pass filter (fourth-order; cut-off frequency 10–500 Hz). The EMG data were then rectified and smoothed by averaging with a sliding window of 30 ms and normalized to the MVC amplitudes (Hellmann et al., 2011).

Based on Posturomed marker data, two critical time points were separately determined for each trial: (1) the start of the perturbation, and (2) the third highest amplitude of the Posturomed center in the direction of perturbation (Kiss, 2011a). The time span between (1) and (2) was assumed to be the phase in which the perturbation was maximally compensated, and the time frames after (2) were considered the dynamic steady-state phase of the movement. For all calculations, a time window of 12 s (Müller et al., 2004) was used which started at time point (2).

### Spatial Dynamics of CoM Sway

To quantify the spatial dynamics of the CoM sway, the PL was calculated. The time series of CoM position was estimated by the subject-specific anthropometric 3D model referenced and explained below (see Section Uncontrolled Manifold Approach). The point-by-point Euclidean norm of the vectors containing the 3D coordinates of the CoM was calculated to convert the three components in the x, y and z coordinates into a single value *k*, where *i* stands for the frame number (Equation 1). PL was approximated by the sum of the distances between consecutive points of the time series *k* with a length of *n* (Prieto et al., 1996), where *n* equals the total number of frames in a 12 s interval (*n* = 2,400; Equation 2).


(1) $$\begin{array}{r} k_{i}=\;\sqrt{{x_{i}}^{2}+\;{y_{i}}^{2}+\;{z_{i}}^{2}};\;where\;i=1,2,3,\ldots ,n \end{array}$$

(2) $$\begin{array}{r} PL=\;\overset{n-1}{\underset{i=1}{\sum }}|k_{i+1}-k_{i}| \end{array}$$

### Temporal Dynamics of CoM Sway

A detrended fluctuation analysis was performed to quantify the temporal dynamics of CoM sway. Firstly, an integrated time series was calculated by subtracting its mean from it (Equation 3). Secondly, the data were divided into non-overlapping segments of length *m* and the linear approximation was estimated by a separate least square fit in each segment. Thirdly, average fluctuation of the time series around the trend was calculated as given in Equation (4). The last two steps were repeated for all the considered *m*.


(3) $$\begin{array}{r} y\left(b\right)=\;\overset{b}{\underset{i=1}{\sum }}\left(k\left(i\right)-k_{avg}\right) \end{array}$$

(4) $$\begin{array}{r} F\left(m\right)=\;\sqrt{\frac{1}{n}\;\overset{n}{\underset{b=1}{\sum }}{\left(y\left(b\right)-y_{m}\left(b\right)\right)}^{2}} \end{array}$$

In general *F*(*m*) increases with the increasing *m* and a power law is expected where the scaling component α is a constant (Equation 5). If α <0.5 or 1 < α <1.5, the time series interpreted as anti-persistent, where a smaller α indicates a more anti-persistent behavior. If 0.5 < α <1 or 1.5 < α <2, the time series is persistent and the larger the α, the more persistent is the time series (Lin et al., 2008).


(5) $$\begin{array}{r} F\left(m\right)\propto m^{\alpha } \end{array}$$

### Uncontrolled Manifold Approach

A UCM approach was applied to investigate if and how the co-variation of the joint movements led to the stabilization and control of the CoM. In accordance with the literature, the CoM and the joint angles were selected as PV and EVs, respectively (Krishnamoorthy et al., 2005; Freitas et al., 2006; Hsu et al., 2007, 2013; Scholz et al., 2007; Hagio et al., 2020). To obtain joint angles, an inverse kinematics calculation was performed using a modified version of the full-body Dynamicus (ALASKA) model (Härtel and Hermsdorf, 2006). A subject-specific anthropometric 3D model was used to estimate the CoM as the weighted sum of the body segments (Möhler et al., 2019).

The model was a modified version of the Hanavan model and had 50 degrees of freedom (Hanavan, 1964). Of the 36 subject-specific anthropometric measurements needed to calculate the CoM according to this model, 21 were taken manually and 15 were determined from the reflective markers. A constant density was assumed (Ackland et al., 1988) and the mass of each segment was estimated by volume integration. The whole-body CoM in 3D, *r*_*CoM*_, was determined by calculating the weighted sum of the body segments using Equation (6), where *N* is the total number of segments (*N* = 17; *V*_*m*_ the volume of the *m*^*th*^ segment; and *r*_*m*_ the center of gravity vector of the *m*^*th*^ segment.


(6) $$\begin{array}{r} r_{CoM\;}=\frac{\;1}{\sum_{m=1}^{N}V_{m}}*\overset{N}{\underset{m=1}{\sum }}r_{m}V_{m} \end{array}$$

The CoM, as the PV for the UCM, was defined as a function of the joint angles as the EVs (*r*_*CoM*_ = *f*(θ_1_, θ_2_, …, θ_*j*_), where j stands for the number of EVs). The mean joint configuration across each trial, θ^0^, was calculated as an approximation of the desired configuration (Latash et al., 2007). The Jacobian matrix, *J*(θ^0^), containing all first-order partial derivatives of the CoM coordinates with respect to the joint angles, was calculated at this reference joint configuration. Afterwards the null space of the matrix was computed as the linear estimate of the UCM (Equation 7). The null space of the Jacobian matrix is the linear subspace of all joint angle combinations that does not affect the position of the CoM, and it is spanned by *j-d* number of basis vectors ε_*i*_, where *j* and *d* are the number of dimensions of EVs and PV, respectively (Scholz and Schöner, 1999).


(7) $$\begin{array}{r} 0=J\left(\theta^{0}\right)\cdot \varepsilon_{i} \end{array}$$

At each instant of *n* = 2,400 trials (*t* = 12 s, recording frequency 200 Hz), the deviation from the mean joint configuration (θ− θ^0^) was calculated (Scholz et al., 2007; Hsu et al., 2013) and subsequently resolved into their projection on the null space to decompose it into the parallel, θ_∥_, and orthogonal, θ_⊥_, components (Scholz and Schöner, 1999; Möhler et al., 2019) (Equations 8, 9).


(8) $$\begin{array}{r} \theta_{\;∥\;}=\;\overset{j-d}{\underset{i=1}{\sum }}\varepsilon_{i}^{T}\;\left(\theta -\;\theta^{0}\right)\;\varepsilon_{i}\; \end{array}$$

(9) $$\begin{array}{r} \theta_{\;⊥\;}=\;\left(\theta -\;\theta^{0}\right)-\;\theta_{\;∥\;} \end{array}$$

Finally, the amount of variability parallel to the UCM (*UCM*_∥_, i.e., stabilizing PV) and orthogonal to the UCM (*UCM*_⊥_, i.e., changing PV) were estimated (Scholz and Schöner, 1999) (Equations 10, 11). *UCM*_*Ratio*_, the ratio of the two UCM components was calculated as suggested by Papi et al. (2015) to obtain a symmetrical distribution (i.e., [−1 1]. The midpoint 0 is the threshold for “synergy” and therefore appropriate for statistical calculations (Equation 12).


(10) $$\begin{array}{r} UCM_{∥}=\sqrt{\frac{1}{n\cdot \left(j-d\right)}\;\overset{n}{\underset{i=1}{\sum }}{\theta_{∥\;i}^{2}}_{\;}} \end{array}$$

(11) $$\begin{array}{r} UCM_{⊥}=\sqrt{\frac{1}{n\cdot \;d\;}\;\overset{n}{\underset{i=1}{\sum }}\theta_{\;⊥\;i}^{2}} \end{array}$$

(12) $$\begin{array}{r} UCM_{Ratio}=\frac{2\cdot {UCM_{∥}}^{2}}{{UCM_{∥}}^{2}+{UCM_{⊥}}^{2}}-1 \end{array}$$

The *UCM*_∥_ component is a measure of the co-variation of EVs and therefore a measure for flexibility. A higher *UCM*_∥_ indicates a higher variability on the level of the EVs that does not change the PV, and therefore a more flexible behavior of the control system, and vice versa. The *UCM*_⊥_ component is a measure for control of the PV. The higher the *UCM*_⊥_ , the less controlled the PV, which in this study is the CoM. Lastly, *UCM*_*Ratio*_ indicates the stability of the PV by means of kinematic synergy of the EVs. A *UCM*_*Ratio*_>0 is interpreted as a synergy, whereas a *UCM*_*Ratio*_ ≤ 0 indicates no synergy (Latash et al., 2007). In this study, *UCM*_*Ratio*_ and *UCM*_⊥_ were used to quantify the stability and control of the CoM (i.e., the PV), respectively.

### Statistics

Statistical analysis was performed with IBM SPSS Statistics 25.0 (IBM Corporation, Armonk, NY, USA). The PL of the CoM, DFA scaling component and two UCM parameters (*UCM*_⊥_, *UCM*_*Ratio*_) for three trials for each direction and for each subject were averaged. A Kolmogorov-Smirnov test was conducted to determine the normality of data distribution.

Each of the four directions of perturbation was analyzed separately because postural response may differ depending on the perturbation direction (Kiss, 2011b; Nonnekes et al., 2013; Chen et al., 2014; Freyler et al., 2015; Akay and Murray, 2021). For each of the four considered parameters and for each direction, a one-way ANOVA or a Kruskal-Wallis test was performed for the group comparisons depending on the normality of the distribution. The level of significance was set *a priori* to *p* < 0.05. Partial eta squared ($\eta_{p}^{2}$) or Cramer's V (ϕ_*c*_) (small effect: $\eta_{p}^{2}$ <0.06 or ϕ_*c*_ <0.2; medium effect: 0.06 < $\eta_{p}^{2}$ <0.14 or 0.2 < ϕ_*c*_ <0.6; large effect: $\eta_{p}^{2}$ > 0.14 or ϕ_*c*_ > 0.6; Cohen, 1988; Richardson, 2011) were calculated to estimate the effect sizes for normal and non-normal distribution of data, respectively.

## Results

### Sway of the Center of Mass

The operationalization of CoM sway in relation to the different stomatognathic motor conditions was analyzed by the PL of the 3D CoM trajectory (Table 2). The PL results did not show any significant changes between different stomatognathic motor conditions in the four perturbation directions. Although B, I and C had small effect sizes, F had a medium effect size (B: *p* = 0.429, $\eta_{p}^{2}$ = 0.037; F: *p* = 0.182, $\eta_{p}^{2}$ = 0.073; I: *p* = 0.461, $\eta_{p}^{2}$ = 0.034; C: *p* = 0.692, $\eta_{p}^{2}$ = 0.016).

**Table 2:** The UCM, the path length and the DFA scaling exponent (α) results are shown as mean ± standard deviation.

**UCM_⊥_in rad^**2**^/dof**	**JAW**	**TON**	**HAB**	***p***	**$\eta_{\text{p}}^{2}$ or ϕ_c_**
B	0.0134 ± 0.0124	0.0125 ± 0.0052	0.0134 ± 0.0064	0.305*	0.157*
F	0.0107 ± 0.0041	0.0127 ± 0.0060	0.0126 ± 0.0052	0.466	0.033
I	0.0173 ± 0.0144	0.0161 ± 0.0085	0.0163 ± 0.0077	0.947	0.002
C	0.0178 ± 0.0113	0.0149 ± 0.0062	0.0179 ± 0.0059	0.514	0.029
**UCM**_**Ratio**_	**JAW**	**TON**	**HAB**	***p***	$\eta_{\text{p}}^{2}$
B	0.2092 ± 0.3179	0.2362 ± 0.2020	0.1852 ± 0.2710	0.865	0.006
F	0.2516 ± 0.3194	0.2204 ± 0.3176	0.1022 ± 0.2482	0.333	0.048
I	0.2492 ± 0.2663	0.2967 ± 0.1652	0.2095 ± 0.2653	0.585	0.024
C	0.1791 ± 0.4335	0.2373 ± 0.2096	0.1634 ± 0.2664	0.788	0.011
**Path length in mm**	**JAW**	**TON**	**HAB**	***p***	$\eta_{\text{p}}^{2}$
B	325.21 ± 174.28	408.17 ± 277.49	329.38 ± 119.33	0.429	0.037
F	267.15 ± 112.13	381.38 ± 253.10	304.44 ± 124.28	0.182	0.073
I	369.18 ± 186.20	423.32 ± 219.60	344.73 ± 125.14	0.461	0.034
C	366.66 ± 154.83	428.83 ± 295.28	395.59 ± 117.34	0.692	0.016
**Scaling component**, **α**	**JAW**	**TON**	**HAB**	***p***	$\eta_{\text{p}}^{2}$
B	1.68 ± 0.12	1.74 ± 0.09	1.76 ± 0.11	0.103	0.096
F	1.73 ± 0.08	1.75 ± 0.09	1.73 ± 0.08	0.724	0.014
I	1.73 ± 0.08	1.72 ± 0.08	1.74 ± 0.09	0.821	0.009
C	1.72 ± 0.09	1.70 ± 0.10	1.71 ± 0.08	0.689	0.016

The p-values and the effect sizes for group comparisons are represented in the last two columns. The asterisks (*) indicate the Kruskal-Wallis and Cramer's V (ϕ_c_) calculations [otherwise a one-way ANOVA and partial eta squared ($\eta_{p}^{\text{2}}$) were calculated]. The level of significance was set a priori to p <0.05. JAW, jaw clenching; TON, tongue pressing; HAB, habitual; B, backwards; F, forwards; I, Ipsilateral; C, contralateral; dof, degrees of freedom.

### Detrended Fluctuation Analysis

Temporal dynamics of CoM sway was analyzed with a DFA (Table 2). The scaling components did not differ significantly between different stomatognathic motor conditions in the four perturbation directions (B: *p* = 0.103, $\eta_{p}^{2}$ = 0.096; F: *p* = 0.724, $\eta_{p}^{2}$ = 0.014; I: *p* = 0.821, $\eta_{p}^{2}$ = 0.009; C: *p* = 0.689, $\eta_{p}^{2}$ = 0.016).

### Uncontrolled Manifold Analysis

A UCM analysis was performed aiming at analyzing the co-variation of joint angles in relation with the control as well as the stability of the CoM. The *UCM*_⊥_ and *UCM*_*Ratio*_ components were utilized to quantify the control and the stability of the CoM, respectively. The results are represented in Table 2.

For the *UCM*_⊥_ component, the groups did not show any significant differences in any of the perturbation directions and all the effect sizes were small (B: *p* = 0.305, ϕ_*c*_ = 0.157; F: *p* = 0.466, $\eta_{p}^{2}$ = 0.033; I: *p* = 0.947, $\eta_{p}^{2}$ = 0.002; C: *p* = 0.514, $\eta_{p}^{2}$ = 0.029). This indicated the control of the CoM was not affected by the stomatognathic motor conditions (i.e., JAW, TON, and HAB).

Regarding the *UCM*_*Ratio*_, the groups did not differ significantly in any of the perturbation directions and all of the results showed small effect sizes (B: *p* = 0.865, $\eta_{p}^{2}$ = 0.006; F: *p* = 0.333, $\eta_{p}^{2}$ = 0.048; I: *p* = 0.585, $\eta_{p}^{2}$ = 0.024; C: *p* = 0.788, $\eta_{p}^{2}$ = 0.011). These results showed that the stability of the CoM was not affected by the stomatognathic motor conditions (i.e., JAW, TON, and HAB).

## Discussion

The aim of this study was to investigate the effects of different stomatognathic motor conditions on the sway, control and stability of the CoM during a dynamic steady-state balance task. The PL of the 3D CoM, a DFA and a UCM analyses were used to quantify the variables of interest. It was hypothesized that jaw clenching and tongue pressing decrease the total sway, increase the persistency of CoM fluctuations, increase both the control and stability of the CoM. Inclusion of the TON group would enable a differentiation between the specific effects of jaw clenching with occlusal load from the effects of stomatognathic motor activity in general, as well as from the dual-task effects. This could ultimately help to understand if the possible modulations of posture during jaw clenching occur basically due to a supra-postural task or stomatognathic activities in general; or if any additional functional interactions such as higher excitability of the human motor system (Boroojerdi et al., 2000), muscle co-contractions (Giannakopoulos et al., 2018a) or H-reflex responses (Miyahara et al., 1996) may exist. None of the considered parameters showed significant group effects in any of the directions. Based on these results, it can be concluded that deliberate jaw clenching or tongue pressing do not have significant effects on the control or stability of the CoM compared to habitual stomatognathic motor conditions in the steady-state phase of the task. At least, the effects could not be quantified by the used parameters. In contrast to the previously-found effects of jaw clenching on dynamic reactive balance performance (Fadillioglu et al., 2022), the findings in this study do not indicate any task-specific effects of stomatognathic motor activities on dynamic steady-state balance assessed by an oscillating platform. Because of the task-specificity of balance (Giboin et al., 2015; Kümmel et al., 2016; Ringhof and Stein, 2018), further research investigating the effects of stomatognathic motor activities on dynamic steady-state balance with different movement tasks are needed. To the best of our knowledge, the present study is the first to investigate the effects of stomatognathic motor activity on dynamic steady-state balance on an oscillating platform.

### Quantification of CoM Sway

The PL of the 3D CoM position was calculated to quantify the distance covered by the CoM during the trials. The results in this study revealed no significant differences between the three groups for any of the directions. Nevertheless, the effect sizes for the direction F were medium (*p* = 0.182; $\eta_{p}^{2}$ = 0.073), where the JAW group had a slightly smaller PL compared to TON and HAB, indicating a higher dynamic steady-state stability. It should be noted that significant performance increases and decreased anatomical region speeds were seen in the F direction during the early reactive phase of the task (Fadillioglu et al., 2022). Although a medium effect size does not have a high statistical power, jaw clenching may have effects on the steady-state stability; but these were not high enough to be detected with the chosen methods.

The temporal dynamics of CoM sway was analyzed by a DFA, which did not show any significant differences between groups. Overall, the scaling exponent α was larger than 1.5 for all directions and all groups, indicating a Brownian noise (Stergiou, 2016). These results were slightly higher but similar to those of Liang et al. (2017) , which considered the CoM instead of center of pressure for DFA (Lin et al., 2008; Blázquez et al., 2010; Munafo et al., 2016) as in the present study. On the other hand, even though DFA has become a predominant method for fractal analysis, it may not provide useful results for short time series (Stergiou, 2016).

### Analysis of Control and Stability of the CoM by a UCM Approach

A UCM approach was applied to investigate the co-variated movement of joints in relation to the CoM position as the PV (Krishnamoorthy et al., 2005; Freitas et al., 2006; Hsu et al., 2007, 2013; Scholz et al., 2007; Hagio et al., 2020) under different stomatognathic motor conditions. In this study, the *UCM*_⊥_ and the *UCM*_*Ratio*_were directly included in the analysis, whereas the *UCM*_∥_ was only indirectly considered within the *UCM*_*Ratio*_. The findings indicate that jaw clenching or tongue pressing do not lead to any effects that are quantifiable with the UCM approach.

The *UCM*_*Ratio*_component was used to investigate the stabilization of the CoM through co-varied movements of the joint angles. The statistical analysis revealed that the three groups did not differ in *UCM*_*Ratio*_. This showed that jaw clenching or tongue pressing did not lead to a more stable CoM compared to habitual stomatognathic motor conditions in the steady-state phase of the task. Therefore, it contradicted our first hypothesis regarding the stability of the CoM.

The *UCM*_⊥_ component was used to quantify the control of the CoM. The results showed that the groups did not differ in terms of control of the CoM, which suggested that jaw clenching or tongue pressing did not lead to a better control compared to habitual conditions. Based on this result, the second hypothesis was rejected.

A UCM analysis was performed in the present study and a subject-specific anthropometric 3D model was used to estimate the CoM (Möhler et al., 2019). Therefore, the model covered all three movement planes and considered not only the lower body but also the upper body, which plays an important role in the movement of the CoM due to its high mass.

### Effects of Masticatory System on Dynamic Stability

As already described in the introduction, there are some phenomena described in the literature that support the assumption of a close functional integration of the masticatory system in the human motor control processes (Miyahara et al., 1996; Boroojerdi et al., 2000; Bracha et al., 2005a,b; Julià-Sánchez et al., 2020). This could be because jaw movements are proportionally driven by anterior neck muscles, which inevitably requires co-contractions of the lateral and posterior neck muscles (Giannakopoulos et al., 2018a). The resulting movements of the head must in turn be matched centrally with the further proprioceptive input of postural control. Therefore, the integration of the stomatognathic system into postural control is not a phenomenon, but a basic requirement.

Jaw clenching during activities that involve the lower and upper limbs may enhance neuromotor stimulation by means of the H-reflex, and therefore increase the excitability of the motor system (de Souza et al., 2021). Furthermore, high activity was observed in the frontal, parietal, and temporal cortices and cerebellum during hand grip combined with jaw clenching compared to without jaw clenching (Kawakubo et al., 2014). In addition, there are also studies revealing that not only stomatognathic activity but also the use of occlusal splints (Battaglia et al., 2018) or mouthguards (Dias et al., 2022) influence the strength in the muscles of the other body parts. These findings indicate that there is a close relationship between the masticatory system and muscle strength or physical exertion. Although it is hard to verify the underlying mechanisms experimentally, based on these findings it was hypothesized that jaw clenching may lead to better dynamic steady-state stability also under dynamic conditions. However, the results in this study did not support this hypothesis.

### Consideration of Methodical Aspects

Based on their gender and baseline performance, the participants were allocated into one of the three groups (JAW, TON, and HAB). The statistical examination by ANOVA revealed that there were no baseline performance differences between the three groups (*p* = 0.767). The purpose of building three groups with different stomatognathic motor conditions, instead of making all participants perform all the tasks, was due to three main reasons. Firstly, “habitual” in this study meant that no instruction was given regarding the stomatognathic motor activity. Therefore, an unconscious, natural behavioral pattern of the masticatory system was ensured. An instructed “habitual” would not be physiologically possible, because an “instructed” behavioral pattern cannot lead to an unconsciously performed behavior. Secondly, possible carry over effects between different stomatognathic motor tasks were avoided. After jaw clenching or tongue pressing, there could be sustained physiological effects such as an increased excitability of the motor system. Thirdly, fatigue effects were avoided. If all tasks were performed for each of the four directions separately, 36 valid trials would be needed. Considering the invalid trials as well, the total number performed would be too high.

In this study, the Posturomed, an oscillating platform, was used to assess dynamic balance performance. The platform was randomly perturbed in one of the four different directions. The perturbation directions were analyzed independently following the suggestions of Freyler et al. (2015), because muscle spindles provide different information depending on the direction as well the velocity of perturbations (Akay and Murray, 2021). In addition to this, the direction of surface translation is an important factor for the sensation, processing and output of the postural responses (Nonnekes et al., 2013; Freyler et al., 2015). Although it was suggested that the perturbation direction may not matter during the steady-state phase of the balancing task on an oscillating platform (Giboin et al., 2015), in this study the directions were analyzed separately since the research question was to further investigate the positive effects of jaw clenching, which was found only in one direction (Fadillioglu et al., 2022).

The focus was put on the CoM in this study because it is suggested to be the controlled variable in postural studies (Winter et al., 1998; Kilby et al., 2015; Nicolai and Audiffren, 2019; Richmond et al., 2021). Also, in studies assessing dynamic stability by means of an oscillating platform, the CoM was considered as an important variable (Pfusterschmied et al., 2013; Pohl et al., 2020). Even if it is widely chosen for postural studies and others from the field of motor control (Scholz et al., 2000, 2001; Reisman et al., 2002; Tseng et al., 2002; Domkin et al., 2005; Verrel et al., 2010; Qu, 2012; Maldonado et al., 2018; Möhler et al., 2019), it does not prove that it is the single right one. Another possible PV could be the distance between the CoM and the center of the platform.

### Limitations

All the participants in this study were physically active adults. The homogeneity of this group helped to minimize altered postural control mechanisms due to, for example, age (Henry and Baudry, 2019) or neurological disorders (Delafontaine et al., 2020). Nevertheless, it should be noted that the findings cannot be directly transferred to other groups (e.g., elders or people with neurological disorders).

The stabilization of a moving platform and the stabilization of the body on a rigid surface are different balance tasks (Alizadehsaravi et al., 2020). Therefore, it should be added that the findings in this study may not be valid for balance tasks on stationary ground or for other dynamic tasks (Giboin et al., 2015; Kümmel et al., 2016; Ringhof and Stein, 2018).

It is possible that the UCM approach and the model used in the study were not sensitive enough to capture the possible effects due the different stomatognathic motor activities. Therefore, further research investigating the effects of stomatognathic motor activities on dynamic steady-state balance with other models could be useful. Additionally, further analysis by use of muscle synergies (d'Avella et al., 2003) or co-contractions (Hellmann et al., 2015; Munoz-Martel et al., 2019) may reveal effects on the level of muscles, which were not visible on the level of kinematics.

## Conclusion

To the best of our knowledge, this study investigates for the first time the effects of different stomatognathic motor conditions (jaw clenching, tongue pressing and habitual condition) on dynamic steady-state balance. The aim was to analyze the effects particularly on the sway, control and stability of the CoM during a dynamic steady-state task (standing one-legged on an oscillating platform). The results revealed that deliberate jaw clenching or tongue pressing do not seem to affect the sway, the control or the stability of the CoM during a dynamic balance task on an oscillating platform. Due to the task-specificity of balance, further research investigating the effects of stomatognathic motor activities on dynamic steady-state balance with different movement tasks is needed. In addition, further analysis by use of muscle synergies or co-contractions may reveal effects on the level of muscles, which were not visible on the level of kinematics. This study can contribute to the understanding of postural control mechanisms, particularly in relation to stomatognathic motor activities.

## Data Availability Statement

The raw data supporting the conclusions of this article will be made available by the authors, without undue reservation.

## Ethics Statement

The studies involving human participants were reviewed and approved by Ethics Committee of the Karlsruhe Institute of Technology. The patients/participants provided their written informed consent to participate in this study. Written informed consent was obtained from the individual(s) for the publication of any potentially identifiable images or data included in this article.

## Author Contributions

CF and LK conducted the experiment. FM provided the toolbox for data processing. CF carried out data analysis and took the lead in writing the manuscript. All authors were involved in the interpretation and discussion of the results, provided critical feedback. All authors contributed to the article and approved the submitted version.

## Funding

This research was supported by the German Research Foundation (STE 2093/4-1 and HE 6961/3-1).

## Conflict of Interest

The authors declare that the research was conducted in the absence of any commercial or financial relationships that could be construed as a potential conflict of interest.

## Publisher's Note

All claims expressed in this article are solely those of the authors and do not necessarily represent those of their affiliated organizations, or those of the publisher, the editors and the reviewers. Any product that may be evaluated in this article, or claim that may be made by its manufacturer, is not guaranteed or endorsed by the publisher.

## References

[B1] Ackland T. R. Henson P. W. Bailey D. A. (1988). The uniform density assumption: its effect upon the estimation of body segment inertial parameters. Int. J. Sport Biomech. 4, 146–155. 10.1123/ijsb.4.2.146

[B2] Akay T. Murray A. J. (2021). Relative contribution of proprioceptive and vestibular sensory systems to locomotion: opportunities for discovery in the age of molecular science. Int. J. Mol. Sci. 22, 1467. 10.3390/ijms22031467 33540567 PMC7867206

[B3] Alghadir A. H. Zafar H. Iqbal Z. A. (2015). Effect of tongue position on postural stability during quiet standing in healthy young males. Somatosens. Motor Res. 32, 183–186. 10.3109/08990220.2015.1043120 26400633

[B4] Alizadehsaravi L. Bruijn S. M. Maas H. van Dieën J. H. (2020). Modulation of soleus muscle H-reflexes and ankle muscle co-contraction with surface compliance during unipedal balancing in young and older adults. Exp. Brain Res. 238, 1371–1383. 10.1007/s00221-020-05784-0 32266445 PMC7286858

[B5] Battaglia G. Messina G. Giustino V. Zangla D. Barcellona M. Iovane A.. (2018). Influence of vertical dimension of occlusion on peak force during handgrip tests in athletes. Asian J. Sports Med. 9, e68274. 10.5812/asjsm.68274

[B6] Bernstein N. A. (1967). The Co-ordination and Regulation of Movements. Oxford: Pergamon Press.

[B7] Blázquez M. T. Anguianoa M. De Saavedra F. A. Lallena A. M. Carpena P. (2010). Characterizing the human postural control system using detrended fluctuation analysis. J. Comput. Appl. Math. 233, 1478–1482. 10.1016/j.cam.2008.04.038

[B8] Boroojerdi B. Battaglia F. Muellbacher W. Cohen L. G. (2000). Voluntary teeth clenching facilitates human motor system excitability. Clin. Neurophysiol. 111, 988–993. 10.1016/S1388-2457(00)00279-0 10825704

[B9] Bracha H. S. Bracha A. S. Williams A. E. Ralston T. C. Matsukawa J. M. (2005b). The human fear-circuitry and fear-induced fainting in healthy individuals–the paleolithic-threat hypothesis. Clin. Auton. Res. 15, 238–241. 10.1007/s10286-005-0245-z 15944875

[B10] Bracha H. S. Ralston T. C. Williams A. E. Yamashita J. M. Bracha A. S. (2005a). The clenching-grinding spectrum and fear circuitry disorders: clinical insights from the neuroscience/paleoanthropology interface. CNS Spectr. 10, 311–318. 10.1017/S109285290002263X 15788958

[B11] Chen C. L. Lou S. Z. Wu H. W. Wu S. K. Yeung K. T. Su F. C. (2014). Effects of the type and direction of support surface perturbation on postural responses. J. Neuroeng. Rehabil. 11, 1–12. 10.1186/1743-0003-11-50 24708582 PMC3986462

[B12] Chen F. C. Stoffregen T. A. (2012). Specificity of postural sway to the demands of a precision task at sea. J. Exp. Psychol. Appl. 18, 203–212. 10.1037/a0026661 22181030

[B13] Cohen J. (1988). Statistical Power Analysis for the Behavioral Sciences. Hillsdale, NJ: Lawrence Erlbaum Associates, 20–27.

[B14] d'Avella A. Saltiel P. Bizzi E. (2003). Combinations of muscle synergies in the construction of a natural motor behavior. Nat. Neurosci. 6, 300–308. 10.1038/nn1010 12563264

[B15] de Souza B. C. Carteri R. B. Lopes A. L. Teixeira B. C. (2021). Teeth clenching can modify the muscle contraction strength of the lower or upper limbs: systematic review. Sport Sci. Health 17, 279–290. 10.1007/s11332-021-00741-y

[B16] Delafontaine A. Hansen C. Marolleau I. Kratzenstein S. Gouelle A. (2020). Effect of a concurrent cognitive task, with stabilizing visual information and withdrawal, on body sway adaptation of Parkinsonian's patients in an off-medication state: a controlled study. Sensors 20, 5059. 10.3390/s20185059 32899926 PMC7571225

[B17] Dias A. Redinha L. Tavares F. Silva L. Malaquias F. Pezarat-Correia P. (2022). The effect of a controlled mandible position mouthguard on upper body strength and power in trained rugby athletes - a randomized within subject study. Injury 53, 457–462. 10.1016/j.injury.2021.11.002 34785082

[B18] Domkin D. Laczko J. Djupsjöbacka M. Jaric S. Latash M. L. (2005). Joint angle variability in 3D bimanual pointing: uncontrolled manifold analysis. Exp. Brain Res. 163, 44–57. 10.1007/s00221-004-2137-1 15668794

[B19] Duarte M. Sternad D. (2008). Complexity of human postural control in young and older adults during prolonged standing. Exp. Brain Res. 191, 265–276. 10.1007/s00221-008-1521-7 18696056

[B20] Dworkin S. F. LeResche L. (1992). Research diagnostic criteria for temporomandibular disorders: review, criteria, examinations and specifications, critique. J. Craniomandib. Disord. Facial Oral Pain 6, 301–355. 1298767

[B21] Ebben W. P. (2006). A brief review of concurrent activation potentiation: theoretical and practical constructs. J. Strength Condit. Res. 20, 985–991. 10.1519/00124278-200611000-00041 17194254

[B22] Ebben W. P. Flanagan E. P. Jensen R. L. (2008). Jaw clenching results in concurrent activation potentiation during the countermovement jump. J. Strength Condit. Res. 22, 1850–1854. 10.1519/JSC.0b013e3181875117 18978622

[B23] Fadillioglu C. Kanus L. Möhler F. Ringhof S. Schindler H. J. Stein T.. (2022). Influence of controlled masticatory muscle activity on dynamic reactive balance. J. Oral Rehabil. 49, 327–336. 10.1111/joor.13284 34811784

[B24] Freyler K. Gollhofer A. Colin R. Brüderlin U. Ritzmann R. (2015). Reactive balance control in response to perturbation in unilateral stance: Interaction effects of direction, displacement and velocity on compensatory neuromuscular and kinematic responses. PLoS ONE 10, e0144529. 10.1371/journal.pone.0144529 26678061 PMC4683074

[B25] Freitas S. M. S. F. Duarte M. Latash M. L. (2006). Two kinematic synergies in voluntary whole-body movements during standing. Journal of Neuro. 95, 636–645. 10.1152/jn.00482.2005 16267118

[B26] Gangloff P. Louis J. P. Perrin P. P. (2000). Dental occlusion modifies gaze and posture stabilization in human subjects. Neurosci. Lett. 293, 203–206. 10.1016/S0304-3940(00)01528-7 11036196

[B27] Gelfand I. M. Latash M. L. (1998). On the problem of adequate language in motor control. Motor Control 2, 306–313. 10.1123/mcj.2.4.306 9758883

[B28] Gera G. Freitas S. Latash M. Monahan K. Schöner G. Scholz J. (2010). Motor abundance contributes to resolving multiple kinematic task constraints. Motor Control 14, 83–115. 10.1123/mcj.14.1.83 20237405 PMC2843002

[B29] Giannakopoulos N. N. Rauer A. K. Hellmann D. Hugger S. Schmitter M. Hugger A. (2018b). Comparison of device-supported sensorimotor training and splint intervention for myofascial temporomandibular disorder pain patients. J. Oral Rehabil. 45, 669–676. 10.1111/joor.12662 29855069

[B30] Giannakopoulos N. N. Schindler H. J. Hellmann D. (2018a). Co-contraction behaviour of masticatory and neck muscles during tooth grinding. J. Oral Rehabil. 45, 504–511. 10.1111/joor.12646 29761534

[B31] Giboin L. S. Gruber M. Kramer A. (2015). Task-specificity of balance training. Hum. Mov. Sci. 44, 22–31. 10.1016/j.humov.2015.08.012 26298214

[B32] Haddad J. M. Rietdyk S. Claxton L. J. Huber J. (2013). Task-dependent postural control throughout the lifespan. Am. College Sports Med. 41, 123–132. 10.1097/JES.0b013e3182877cc8 23364347 PMC3608710

[B33] Hagio K. Obata H. Nakazawa K. (2020). Effects on postural kinematics of performing a cognitive task during upright standing. Percept. Mot. Skills 127, 639–650. 10.1177/0031512520919543 32340552

[B34] Hanavan E. P. J. (1964). A Mathematical Model of the Human Body. (AMRL-TR-64). Ohio: Aerospace Medical Research Laboratories. 14243640

[B35] Härtel T. Hermsdorf H. (2006). Biomechanical modelling and simulation of human body by means of DYNAMICUS. J. Biomech. 39, 549. 10.1016/S0021-9290(06)85262-0

[B36] Hellmann D. Giannakopoulos N. N. Blaser R. Eberhard L. Schindler H. J. (2011). The effect of various jaw motor tasks on body sway. J. Oral Rehabil. 38, 729–736. 10.1111/j.1365-2842.2011.02211.x 21385200

[B37] Hellmann D. Stein T. Potthast W. Rammelsberg P. Schindler H. J. Ringhof S. (2015). The effect of force-controlled biting on human posture control. Hum. Mov. Sci. 43, 125–137. 10.1016/j.humov.2015.08.009 26282375

[B38] Henry M. Baudry S. (2019). Age-related changes in leg proprioception: implications for postural control. J. Neurophysiol. 122, 525–538. 10.1152/jn.00067.2019 31166819 PMC6734411

[B39] Hermens H. Freriks B. Merletti R. Stegeman D. Blok J. Rau G.. (1999). European Recommendations for Surface Electromyography: Results of the SENIAM Project. Enschede: Roessingh Research and Development.

[B40] Horak F. B. (2006). Postural orientation and equilibrium: what do we need to know about neural control of balance to prevent falls? Age Ageing 35, 7–11. 10.1093/ageing/afl077 16926210

[B41] Hrysomallis C. (2007). Relationship between balance ability, training and sports injury risk. Sports Med. 37, 547–556. 10.2165/00007256-200737060-00007 17503879

[B42] Hsu W. L. Chou L. S. Woollacott M. (2013). Age-related changes in joint coordination during balance recovery. Age 35, 1299–1309. 10.1007/s11357-012-9422-x 22618298 PMC3705105

[B43] Hsu W. L. Scholz J. P. Schöner G. Jeka J. J. Kiemel T. (2007). Control and estimation of posture during quiet stance depends on multijoint coordination. J. Neurophysiol. 97, 3024–3035. 10.1152/jn.01142.2006 17314243

[B44] Jendrassik E. (1885). Zur Untersuchung des Kniephaenomens. Neur Zbl. 4, 412–415.

[B45] Julià-Sánchez S. Álvarez-Herms J. Cirer-Sastre R. Corbi F. Burtscher M. (2020). The influence of dental occlusion on dynamic balance and muscular tone. Front. Physiol. 10, 1626. 10.3389/fphys.2019.01626 32082183 PMC7005008

[B46] Kawakubo N. Miyamoto J. J. Katsuyama N. Ono T. Honda E. ichi Kurabayashi T.. (2014). Effects of cortical activations on enhancement of handgrip force during teeth clenching: an fMRI study. Neurosci. Res. 79, 67–75. 10.1016/j.neures.2013.11.006 24326095

[B47] Kilby M. C. Molenaar P. C. M. Newell K. M. (2015). Models of postural control: shared variance in joint and COM motions. PLoS ONE 10, e0126379. 10.1371/journal.pone.0126379 25973896 PMC4431684

[B48] Kiss R. M. (2011a). A new parameter for characterizing balancing ability on an unstable oscillatory platform. Med. Eng. Phys. 33, 1160–1166. 10.1016/j.medengphy.2011.04.017 21616699

[B49] Kiss R. M. (2011b). Do lateral dominance, body mass, body height and direction of perturbation influence the lehr's damping ratio, which characterizes the balancing ability on an unstable oscillatory platform? WIT Trans. Biomed. Health 15, 373–382. 10.2495/EHR110321

[B50] Krishnamoorthy V. Yang J. F. Scholz J. P. (2005). Joint coordination during quiet stance: effects of vision. Exp. Brain Res. 164, 1–17. 10.1007/s00221-004-2205-6 15841397

[B51] Kümmel J. Kramer A. Giboin L. S. Gruber M. (2016). Specificity of balance training in healthy individuals: a systematic review and meta-analysis. Sports Med. 46, 1261–1271. 10.1007/s40279-016-0515-z 26993132

[B52] Latash M. L. Scholz J. P. Schöner G. (2002). Motor control strategies revealed in the structure of motor variability. Exerc. Sport Sci. Rev. 30, 26–31. 10.1097/00003677-200201000-00006 11800496

[B53] Latash M. L. Scholz J. P. Schöner G. (2007). Toward a new theory of motor synergies. Motor Control 11, 276–308. 10.1123/mcj.11.3.276 17715460

[B54] Liang H. Beerse M. Ke X. Wu J. (2017). Effect of whole-body vibration on center-of-mass movement during standing in children and young adults. Gait Posture 54, 148–153. 10.1016/j.gaitpost.2017.03.005 28292716

[B55] Lin D. Seol H. Nussbaum M. A. Madigan M. L. (2008). Reliability of COP-based postural sway measures and age-related differences. Gait Posture 28, 337–342. 10.1016/j.gaitpost.2008.01.005 18316191

[B56] Maldonado G. Bailly F. Souères P. Watier B. (2018). On the coordination of highly dynamic human movements: an extension of the Uncontrolled Manifold approach applied to precision jump in parkour. Sci. Rep. 8, 1–14. 10.1038/s41598-018-30681-6 30111843 PMC6093881

[B57] Miyahara T. Hagiya N. Ohyama T. Nakamura Y. (1996). Modulation of human soleus H reflex in association with voluntary clenching of the teeth. J. Neurophysiol. 76, 2033–2041. 10.1152/jn.1996.76.3.2033 8890312

[B58] Mochida Y. Yamamoto T. Fuchida S. Aida J. Kondo K. (2018). Does poor oral health status increase the risk of falls?: the JAGES project longitudinal study. PLoS ONE 13, e0192251. 10.1371/journal.pone.0192251 29389975 PMC5794168

[B59] Möhler F. Ringhof S. Debertin D. Stein T. (2019). Influence of fatigue on running coordination: a UCM analysis with a geometric 2D model and a subject-specific anthropometric 3D model. Hum. Mov. Sci. 66, 133–141. 10.1016/j.humov.2019.03.016 30981149

[B60] Müller O. Günther M. Krau,ß I. Horstmann T. (2004). Physikalische Charakterisierung des Therapiegerätes Posturomed als Meßgerät - Vorstellung eines Verfahrens zur Quantifizierung des Balancevermögens. Biomedizinische Technik 49, 56–60. 10.1515/BMT.2004.011 15106899

[B61] Munafo J. Curry C. Wade M. G. Stoffregen T. A. (2016). The distance of visual targets affects the spatial magnitude and multifractal scaling of standing body sway in younger and older adults. Exp. Brain Res. 234, 2721–2730. 10.1007/s00221-016-4676-7 27255223

[B62] Munoz-Martel V. Santuz A. Ekizos A. Arampatzis A. (2019). Neuromuscular organisation and robustness of postural control in the presence of perturbations. Sci. Rep. 9, 1–10. 10.1038/s41598-019-47613-7 31439926 PMC6706387

[B63] Nicolai A. Audiffren J. (2019). “Estimating center of mass trajectory in quiet standing: a review,” in Proceedings of the Annual International Conference of the IEEE Engineering in Medicine and Biology Society. EMBS, 6854–6859. 31947415 10.1109/EMBC.2019.8857888

[B64] Nonnekes J. Scotti A. Oude Nijhuis L. B. Smulders K. Queralt A. Geurts A. C. H.. (2013). Are postural responses to backward and forward perturbations processed by different neural circuits? Neuroscience 245, 109–120. 10.1016/j.neuroscience.2013.04.036 23624061

[B65] Okubo M. Fujinami Y. Minakuchi S. (2010). The effect of complete dentures on body balance during standing and walking in elderly people. J. Prosthodont. Res. 54, 42–47. 10.1016/j.jpor.2009.09.002 19819207

[B66] Papi E. Rowe P. J. Pomeroy V. M. (2015). Analysis of gait within the uncontrolled manifold hypothesis: stabilisation of the centre of mass during gait. J. Biomech. 48, 324–331. 10.1016/j.jbiomech.2014.11.024 25488137

[B67] Pfusterschmied J. Buchecker M. Keller M. Wagner H. Taube W. Müller E. (2013). Supervised slackline training improves postural stability. Eur. J. Sport Sci. 13, 49–57. 10.1080/17461391.2011.583991

[B68] Pohl T. Brauner T. Wearing S. Horstmann T. (2020). Limb movement, coordination and muscle activity during a cross-coordination movement on a stable and unstable surface. Gait Posture 81, 131–137. 10.1016/j.gaitpost.2020.07.019 32888551

[B69] Prieto T. E. Myklebust J. B. Hoffmann R. G. Lovett E. G. Myklebust B. M. (1996). Measures of postural steadiness: differences between healthy young and elderly adults. IEEE Trans. Biomed. Eng. 43, 956–966. 10.1109/10.532130 9214811

[B70] Qu X. (2012). Uncontrolled manifold analysis of gait variability: effects of load carriage and fatigue. Gait Posture 36, 325–329. 10.1016/j.gaitpost.2012.03.004 22464638

[B71] Reisman D. S. Scholz J. P. Schöner G. (2002). Coordination underlying the control of whole body momentum during sit-to-stand. Gait Posture 15, 45–55. 10.1016/S0966-6362(01)00158-8 11809580

[B72] Richardson J. T. E. (2011). Eta squared and partial eta squared as measures of effect size in educational research. Educ. Res. Rev. 6, 135–147. 10.1016/j.edurev.2010.12.001

[B73] Richmond S. B. Fling B. W. Lee H. Peterson D. S. (2021). The assessment of center of mass and center of pressure during quiet stance: current applications and future directions. J. Biomech. 123, 110485. 10.1016/j.jbiomech.2021.110485 34004395

[B74] Ringhof S. Leibold T. Hellmann D. Stein T. (2015b). Postural stability and the influence of concurrent muscle activation - beneficial effects of jaw and fist clenching. Gait Posture 42, 598–600. 10.1016/j.gaitpost.2015.09.002 26385200

[B75] Ringhof S. Stein T. (2018). Biomechanical assessment of dynamic balance: specificity of different balance tests. Hum. Mov. Sci. 58, 140–147. 10.1016/j.humov.2018.02.004 29438911

[B76] Ringhof S. Stein T. Hellmann D. Schindler H. J. Potthast W. (2016). Effect of jaw clenching on balance recovery: dynamic stability and lower extremity joint kinematics after forward loss of balance. Front. Psychol. 7, 291. 10.3389/fpsyg.2016.00291 27014116 PMC4786560

[B77] Ringhof S. Stein T. Potthast W. Schindler H. J. Hellmann D. (2015a). Force-controlled biting alters postural control in bipedal and unipedal stance. J. Oral Rehabil. 42, 173–184. 10.1111/joor.12247 25354425

[B78] Rubenstein L. Z. (2006). Falls in older people: epidemiology, risk factors and strategies for prevention. Age Ageing 35, 37–41. 10.1093/ageing/afl084 16926202

[B79] Sakaguchi K. Mehta N. R. Abdallah E. F. Forgione A. G. Hirayama H. Kawasaki T.. (2007). Examination of the relationship between mandibular position and body posture. Cranio 25, 237–249. 10.1179/crn.2007.037 17983123

[B80] Schmidt R. A. Lee T. D. Winstein C. J. Wulf G. Zelaznik H. N. (2018). Motor Control and Learning: A Behavioral Emphasis. 6th Edn. Champaign: Human Kinetics.

[B81] Scholz J. P. Reisman D. Schöner G. (2001). Effects of varying task constraints on solutions to joint coordination in a sit-to-stand task. Exp. Brain Res. 141, 485–500. 10.1007/s002210100878 11810142

[B82] Scholz J. P. Schöner G. (1999). The uncontrolled manifold concept: identifying control variables for a functional task. Exp. Brain Res. 126, 289–306. 10.1007/s002210050738 10382616

[B83] Scholz J. P. Schöner G. (2014). “Use of the uncontrolled manifold (UCM) approach to understand motor variability, motor equivalence, and self-motion,” in Progress in Motor Control, ed M. F. Levin (New York, NY: Springer), 91–100. 10.1007/978-1-4939-1338-1_7 25330887

[B84] Scholz J. P. Schöner G. Hsu W. L. Jeka J. J. Horak F. Martin V. (2007). Motor equivalent control of the center of mass in response to support surface perturbations. Exp. Brain Res. 180, 163–179. 10.1007/s00221-006-0848-1 17256165

[B85] Scholz J. P. Schöner G. Latash M. L. (2000). Identifying the control structure of multijoint coordination during pistol shooting. Exp. Brain Res. 135, 382–404. 10.1007/s002210000540 11146817

[B86] Shumway-Cook A. Woollacott M. (2017). Motor control. Translating Research into Clinical Practice. 5th Edn. Philadelphia, PA: Wolters Kluwer.

[B87] Stergiou N. (2016). Nonlinear Analysis for Human Movement Variability. 1st Edn. Boca Raton, FL: CRC Press.

[B88] Stetter B. J. Herzog M. Möhler F. Sell S. Stein T. (2020). Modularity in motor control: similarities in kinematic synergies across varying locomotion tasks. Front. Sports Active Living 2, 596063. 10.3389/fspor.2020.596063 33345175 PMC7739575

[B89] Takakusaki K. (2017). Functional neuroanatomy for posture and gait control. J. Mov. Disord. 10, 1–17. 10.14802/jmd.16062 28122432 PMC5288669

[B90] Todorov E. Jordan M. I. (2002). Optimal feedback control as a theory of motor coordination. Nat. Neurosci. 5, 1226–1235. 10.1038/nn963 12404008

[B91] Tseng Y. Scholz J. P. Schöner G. (2002). Goal-equivalent joint coordination in pointing: affect of vision and arm dominance. Motor Control 6, 183–207. 10.1123/mcj.6.2.183 12122226

[B92] Verrel J. Lövdén M. Lindenberger U. (2010). Motor-equivalent covariation stabilizes step parameters and center of mass position during treadmill walking. Exp. Brain Res. 207, 13–26. 10.1007/s00221-010-2424-y 20862457

[B93] Winter D. A. Patla A. E. Prince F. Ishac M. Gielo-perczak K. (1998). Stiffness control of balance in quiet standing. J. Neurophysiol. 80, 1211–1221. 10.1152/jn.1998.80.3.1211 9744933

